# Automated NMR relaxation dispersion data analysis using NESSY

**DOI:** 10.1186/1471-2105-12-421

**Published:** 2011-10-27

**Authors:** Michael Bieri, Paul R Gooley

**Affiliations:** 1 Department of Biochemistry and Molecular Biology, Bio21 Molecular Science and Biotechnology Institute, University of Melbourne, Parkville, Vic 3010, Australia

**Keywords:** Protein dynamics, software, cpmg, conformational/chemical exchange, μs-ms motion, van't Hoff, transition state theory

## Abstract

**Background:**

Proteins are dynamic molecules with motions ranging from picoseconds to longer than seconds. Many protein functions, however, appear to occur on the micro to millisecond timescale and therefore there has been intense research of the importance of these motions in catalysis and molecular interactions. Nuclear Magnetic Resonance (NMR) relaxation dispersion experiments are used to measure motion of discrete nuclei within the micro to millisecond timescale. Information about conformational/chemical exchange, populations of exchanging states and chemical shift differences are extracted from these experiments. To ensure these parameters are correctly extracted, accurate and careful analysis of these experiments is necessary.

**Results:**

The software introduced in this article is designed for the automatic analysis of relaxation dispersion data and the extraction of the parameters mentioned above. It is written in Python for multi platform use and highest performance. Experimental data can be fitted to different models using the Levenberg-Marquardt minimization algorithm and different statistical tests can be used to select the best model. To demonstrate the functionality of this program, synthetic data as well as NMR data were analyzed. Analysis of these data including the generation of plots and color coded structures can be performed with minimal user intervention and using standard procedures that are included in the program.

**Conclusions:**

NESSY is easy to use open source software to analyze NMR relaxation data. The robustness and standard procedures are demonstrated in this article.

## Background

Proteins are responsible for many different and important biochemical functions, such as ligand binding, signaling or catalysis [1-4]. Many of these events occur on the μs-ms timescale making the detection and interpretation of these motions critical for understanding their biological significance. Advances in solution Nuclear Magnetic Resonance (NMR) of biomolecules have enabled the measurement of protein dynamics on different timescales. Relaxation experiments measuring the two relaxation rates R_1 _(the longitudinal relaxation rate) and R_2 _(the transverse relaxation rate) as well as the steady state heteronuclear NOE are used to detect motion within ps-ns [5]. However, detection of these motions is limited by the molecular tumbling of the molecule, which is on the order of ns. Recent developments in ^15^N/^13^C relaxation-compensated Carr-Purcell-Meiboom-Gill (CPMG) and R_1ρ _rotating-frame relaxation dispersion experiments [6-9] have enabled measurement of protein dynamics on the μs-ms timescale. From these experiments the exchange contribution to transverse relaxation (*R_ex_*), exchange constant (*k_ex_*), chemical shift differences between exchanging states (*δω*) and the population of individual states can be extracted. Consequently, relaxation dispersion experiments are commonly used to investigate changes in dynamics caused by ligand binding [10], to discover limiting events during enzyme catalysis [8], to follow protein folding and intermediate states as well as to detect functionally important hidden states [11]. Furthermore, it has been proposed that a detailed understanding of the dynamical behavior of a protein at atomic resolution may help identify novel drug target sites [12].

Here, we present a novel software (NESSY) that is designed for simple and automated analysis of relaxation dispersion data. Data can be fitted to two states and at one or more field strengths. Model selection protocols are included for the selection of the best models and Monte Carlo simulations are used to estimate errors of extracted parameters. Furthermore, publication quality 2D and 3D plots and color coded structures are easily created.

## Implementation

### Programming

**Figure 1:**
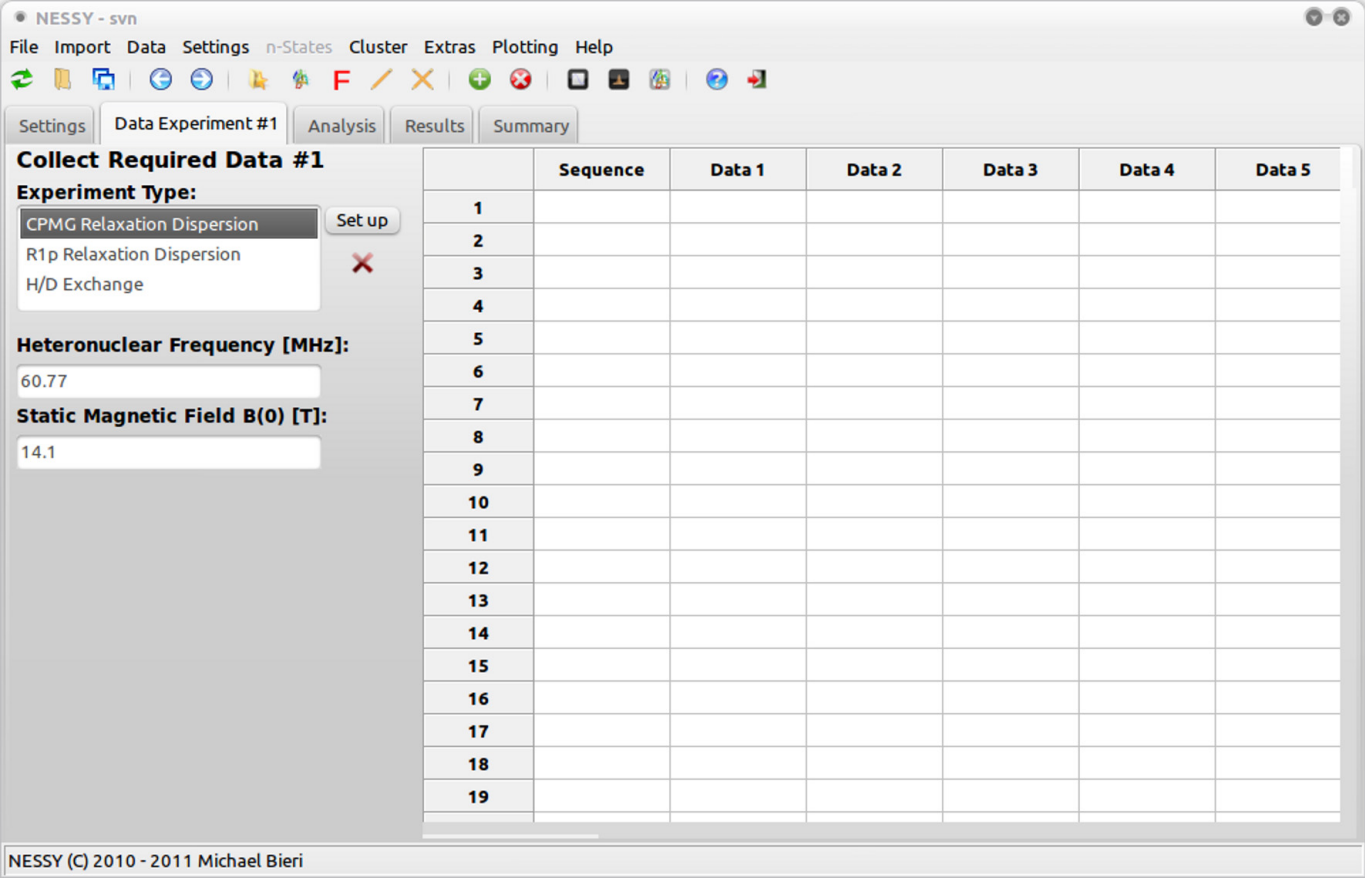
**The graphical user interface of NESSY**. Data is imported into the interface of NESSY and analysis can be followed in real time in the Start Analysis section. Results are summarized and grouped in the Results and Summary section.

The software NESSY is written in Python http://www.python.org for multi platform compatibility and uses wx.Python http://www.wxpython.org to build the graphical user interface (Figure 1). The program was tested on Windows (Vista and 7) and Linux (Ubuntu 9.10 to 11.04) and can be downloaded for free from http://nessy.biochem.unimelb.edu.au either as compiled binaries (Linux, Mac or Windows format, including all Python modules) or as source code. The software is open source and general public licensed (GPL v3).

### Theory

NESSY performs automatic relaxation dispersion data analysis using NMR peak lists as inputs. In a first step, the effective transverse relaxation rate R_2_^eff ^is extracted from a series of CPMG relaxation dispersion experiments according to:

(1) R2eff=1/TCPMG lnI(0)/I(vCPMG)

where *T_CPMG _*is the constant CPMG time, *I(0) *is the intensity of the peak in the reference spectrum and *I(v_CPMG_) *is the intensity of the peak at the CPMG frequency, *v*_CPMG_. Then, data are fitted to different models, which can be selected individually. These models are divided on the basis of no exchange (model 1), two-site fast (model 2) and two-site slow (model 3) exchange.

### No exchange: model 1

Model 1 describes residues, which are not involved in exchange processes:

(2) $$R_{2}^{eff}=R_{2}^{0}$$

where $R_{2}^{0}$ is the effective transverse relaxation rate at infinite *v*_CPMG_.

### Two-state exchange: model 2 and 3

Equation (3) describes exchange between two states A and B

(3) $$A\;\overset{k_{a-b}}{\underset{k_{b-a}}{⇌}}\;B$$

Where *k_a-b _*and *k_b-a _*are the forward and backward exchange rates, respectively, between the states A and B.

Model 2 describes fast-limit exchange (k_ex _>> δω) and is fitted to [7,13]:

(4) $$R_{2}^{eff}=R_{2}^{0}+\frac{\Phi }{k_{ex}}\left[1-\frac{4v_{CPMG}}{k_{ex}}tanh\left(\frac{k_{ex}}{4v_{CPMG}}\right)\right]$$

where

$$\Phi =p_{a}*p_{b}*\delta \omega^{\text{2}}$$

and *p_a _*and *p_b _*are the populations of the two state models (*p_a _*is the major conformation, *p_a _*+ *p_b _*= 1), *k*_*e*x _is the chemical/conformational exchange (*k*_ex _= *k_a-b _*+ *k_b-a_*) constant and *δω *is the chemical shift difference between states. In eq. (4) only $R_{2}^{0}$, *k_ex _*and *Φ *can be extracted, as *p_b _*and, *δω *cannot be uniquely determined.

Model 3 describes slow-limit exchange (k_ex _<< δω) according to the Richard-Carver equation [14]:

(5) $$\begin{array}{c} R_{2}^{eff}=R_{2}^{0}+\frac{k_{ex}}{2}\\ -v_{CPMG}cosh^{-1}\left[D_{+}\cosh\left(\eta_{+}\right)-D_{-}\cos\left(\eta_{-}\right)\right] \end{array}$$

where

$$\begin{array}{c} D_{\pm }=\frac{1}{2}\left[\pm 1+\frac{\Psi +2\delta \omega^{2}}{{\left(\Psi^{2}+\xi^{2}\right)}^{1/2}}\right]\\ \eta_{\pm }=\frac{{\left[\pm \Psi +{\left(\Psi^{2}+\xi^{2}\right)}^{1/2}\right]}^{1∕2}}{2\sqrt{2}v_{CPMG}}\\ \Psi ={k_{ex}}^{2}-\delta \omega^{2}\\ \xi =-2\delta \omega \left(p_{a}k_{ex}-p_{b}k_{ex}\right) \end{array}$$

NESSY also automatically calculates the exchange contribution to transverse relaxation, *R_ex_*, where for fast exchange, *R_ex _= Φ*/*k_ex_*, and slow exchange, *R_ex _*= *p_a_p_b_k_ex_*/*(1 + (k_ex_/δω)^2^)*.

### Optimization and Model Selection

Exchange parameters are optimized by using the Levenberg-Marquardt algorithm to minimize the target function

(6) $$\chi^{2}=\sum_{n}\frac{{\left(R_{2}^{n,eff,calc}-R_{2}^{n,eff}\right)}^{2}}{{\left(\sigma_{R_{2}^{eff}}\right)}^{2}}$$

where $R_{2}^{n,eff}$ is the value at each *v_CPMG _*at data point *n*, $R_{2}^{n,eff,calc}$ is the calculated rate constant. Error $\sigma_{R_{2}^{eff}}$ of *R*_2_*^eff ^*was assumed to be constant within each residue and calculated according to the definition of pooled variance (eq. 7) [15]. $\sigma_{R_{2}^{eff}}$ is calculated using *N_dup _*replicated experiments for *v_CPMG _*values, such that *n*_*j *_replicates were obtained at (*v_CPMG_*)_j_. The effective transverse relaxation rate of a given peak $R_{2}^{eff}$ in these *n_j _*replicates has the standard deviation *s_j_*:

(7) $$\sigma_{R_{2}^{eff}}^{2}=\frac{\overset{N_{dup}}{\underset{j=1}{\sum }}s_{j}^{2}\left(n_{j}-1\right)}{\overset{N_{dup}}{\underset{j=1}{\sum }}\left(n_{j}-1\right)}$$

Model selection is performed using AICc (Akaike information criteria with second order correction for small sample size) by default, but other tests, such as AIC or F-test are included as well [16]. Uncertainties are estimated using 500 (default value, but user controlled) Monte Carlo simulations.

### Synthetic data

To demonstrate validity of fit and model selection performed by NESSY, synthetic data was produced using the in-built Synthetic Data Creator. Data were created for models 2 and 3 by introducing an error of 2, 5, 8 and 10% (see Table 1) as experimental NMR relaxation dispersion data usually have smaller errors than 5%. Data was analyzed using NESSY and fitted to the models described above.

**Table 1:** Extracted dynamics parameters of synthetic relaxation dispersion data.

	Model	R_2 _[1/s]	err	k_ex _[1/s]	err	p_b_	err	Φ	δω
Model 2, original	2	15.23		3750.3				47457.4	
Model 2, ± 2%	2	14.93	0.06	4015.0	34.8			51506.0	
Model 2, ± 5%	2	15.49	0.29	3742.5	188.1			46250.6	
Model 2, ± 8%	2	15.11	0.4	3795.6	251.8			48212.4	
Model 2, ± 10%	2	14.35	0.24	4303.7	148.4			57796.3	
									
Model 3, original	3	15.23		306.2		0.072			1875.5
Model 3, ± 2%	3	15.18	0.03	330.8	21.8	0.067	0.004		1874.8
Model 3, ± 5%	3	15.18	0.09	330.3	64.4	0.068	0.017		1921.0
Model 3, ± 8%	3	15.67	0.32	231.7	152.0	0.092	0.110		1800.0
Model 3, ± 10%	3	15.52	0.33	363.9	170.4	0.060	0.082		1767.5

### NMR spectroscopy

Carr-Purcell-Meiboom-Gill (CPMG) relaxation dispersion experiments of a 0.3 mM PCTX1 sample (kindly provided by Prof. G. King, University of Queensland, Australia) were recorded on a Bruker 600 MHz AVANCE III spectrometer. Experiments were acquired at 298 K using 0.08 s constant CPMG period (T_CPMG_) as a single scanned interleaved pseudo 3D experiment. Relaxation dispersion profiles were obtained by recording spectra with varying CPMG pulse frequencies (ν_CPMG_) (25, 2× 50, 75, 100, 2× 150, 2× 200, 300, 500, 600, 700, 900, 1000, 1500 and 2000 Hz). Spectra were processed in NMRPipe [17] and intensities extracted using CcpNmr [18].

## Results

### Synthetic data

**Figure 2:**
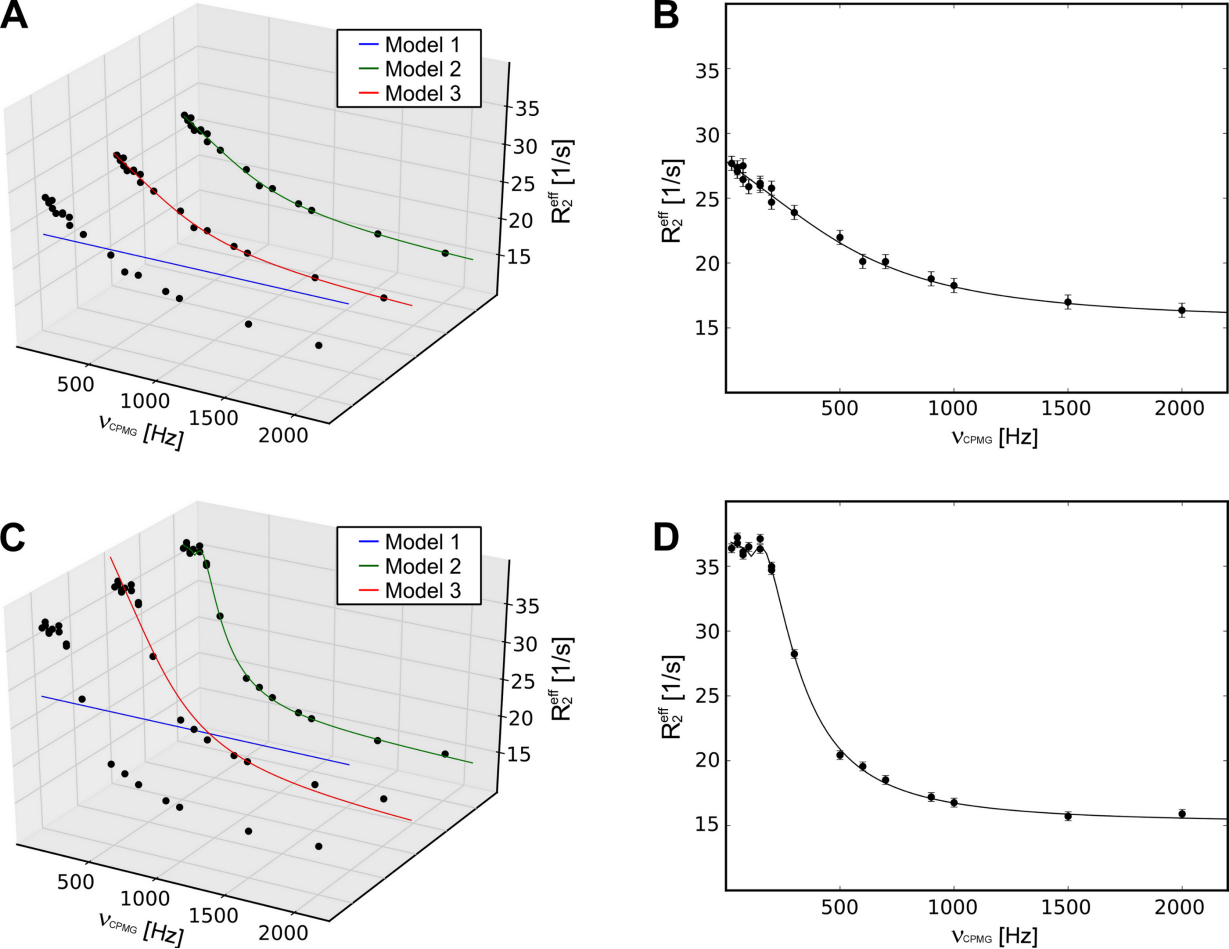
**Comparison of fits of models 1 to 3 to synthetic data**. (A) Individual fits of models 1 to 3 to the synthetic data for model 2 with 5% introduced error and (B) best fit (model 2). (C) Overview of fits (model 1 to 3) to synthetic data for model 3 with 5% introduced error. Best fit is displayed separately (D).

To demonstrate the validity of the coding and model selection, synthetic data containing 2, 5, 8 and 10% errors for models 2 and 3 were created using the in-built tool in NESSY. This synthetic data was fitted to models 1, 2 and 3 by minimizing the χ^2 ^function (eq. 6) and the model selection was performed by comparing AICc values. Errors for the extracted dynamics parameters were estimated by 500 Monte Carlo simulations. In all cases, irrespective of the noise, the correct model was chosen by AICc for each data set (Tables 1 and 2). Notably, for the synthetic-data model 2 the χ^2 ^values calculated for model 3 were always at least as good as those for model 2, indicating the need for model selection by statistical methods such as AICc. For the synthetic-data model 3, both χ^2 ^and AICc selected model 3. During the calculation, NESSY creates plots for each fit to a model. For example, a comparison of fits to models 1, 2 and 3 for the synthetic data is shown in Figure 2 (3D plots are generated using the NESSY plotting tool).

**Table 2:** Model selection of synthetic relaxation dispersion data.

Synthetic Data	Model 1		Model 2		Model 3		Selection
	χ^2^	AICc	χ^2^	AICc	χ^2^	AICc	
		
Model 2, 2% error	29742.68	29744.68	24.75	34.75	24.77	38.77	2
Model 2, 5% error	912.17	914.17	7.68	17.68	7.64	21.64	2
Model 2, 8% error	535.24	537.24	9.63	19.63	9.63	23.63	2
Model 2, 10% error	2001.9	2003.9	19.78	29.78	19.78	33.78	2
Model 3, 2% error	88082.77	88084.77	2721.03	2731.03	18.26	32.26	3
Model 3, 5% error	12471.09	12473.09	445.54	455.54	22.36	36.36	3
Model 3, 8% error	915.07	917.07	38.98	48.98	5.4	19.4	3
Model 3, 10% error	921.28	923.28	27.61	37.61	5.66	19.66	3

The parameters *k_ex_, p_b _*and *δω *that were extracted for model 3 matched the initial parameters used to generate the synthetic data within the order of the introduced error (Table 1). As expected, introducing larger errors into the synthetic data sets produced larger errors for the extracted parameters (calculated by Monte Carlo Simulations).

### Fitting relaxation dispersion experiments of PCTX1 at 600 MHz

**Figure 3:**
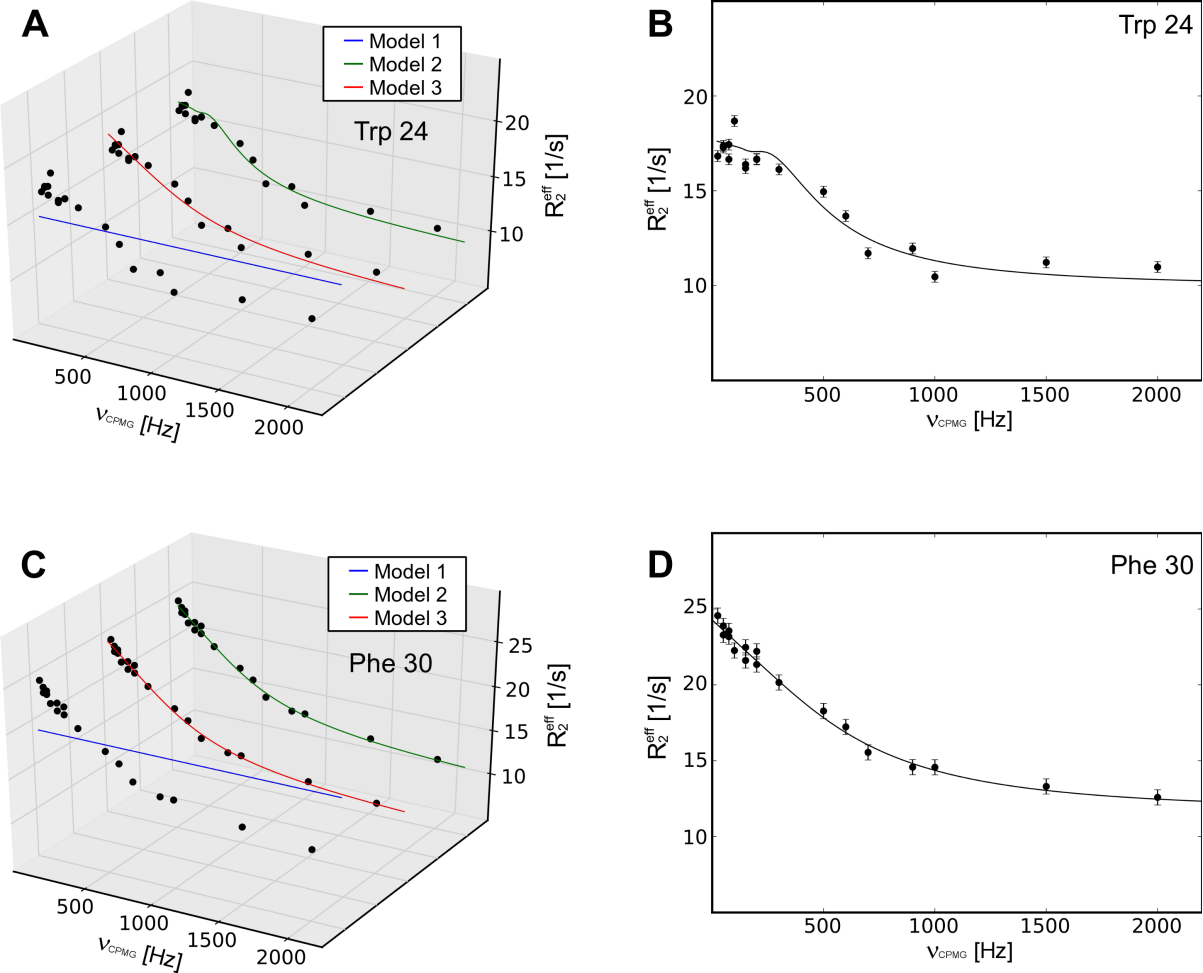
**Analysis of 2 signals of PCTX1**. Residue Trp 24 best fits to a slow exchange 2-state model, (model 3) (A and B), whereas Phe 30 was best described by a fast exchange 2-state model (model 2) (C and D).

NESSY was tested using NMR data acquired at 600 MHz for PCTX1, a 40-residue spider toxin [19]. Data for two resonances showing frequency dependent *R_ex _*were fitted to models 1 to 3. Trp 24 best fitted to model 3 (two-state, slow-limit exchange) and Phe 30 to model 2 (two-state, fast-limit exchange). An overview of fits to model 1 to 3 as well as the best fit is shown in Figure 3. Fitting of the slow-limit exchanging signal of Trp 24 extracts a *k_ex _*of 1029.3 ± 194 1/s. Although the fit shows slow-limit exchange, only one resonance was present in the spectrum. This is in accordance to the extracted population of the minor conformation (*p_b_*) of 0.844 ± 0.002% and a chemical shift difference (*δω*) for the two conformations of 2884 ± 76 rad/s. As residue Phe 30 is in fast exchange, only *k_ex _*and *Φ *were extracted. The extracted conformation exchange rate (*k_ex_*) for this residue was 3750.2 ± 162 1/s.

**Figure 4:**
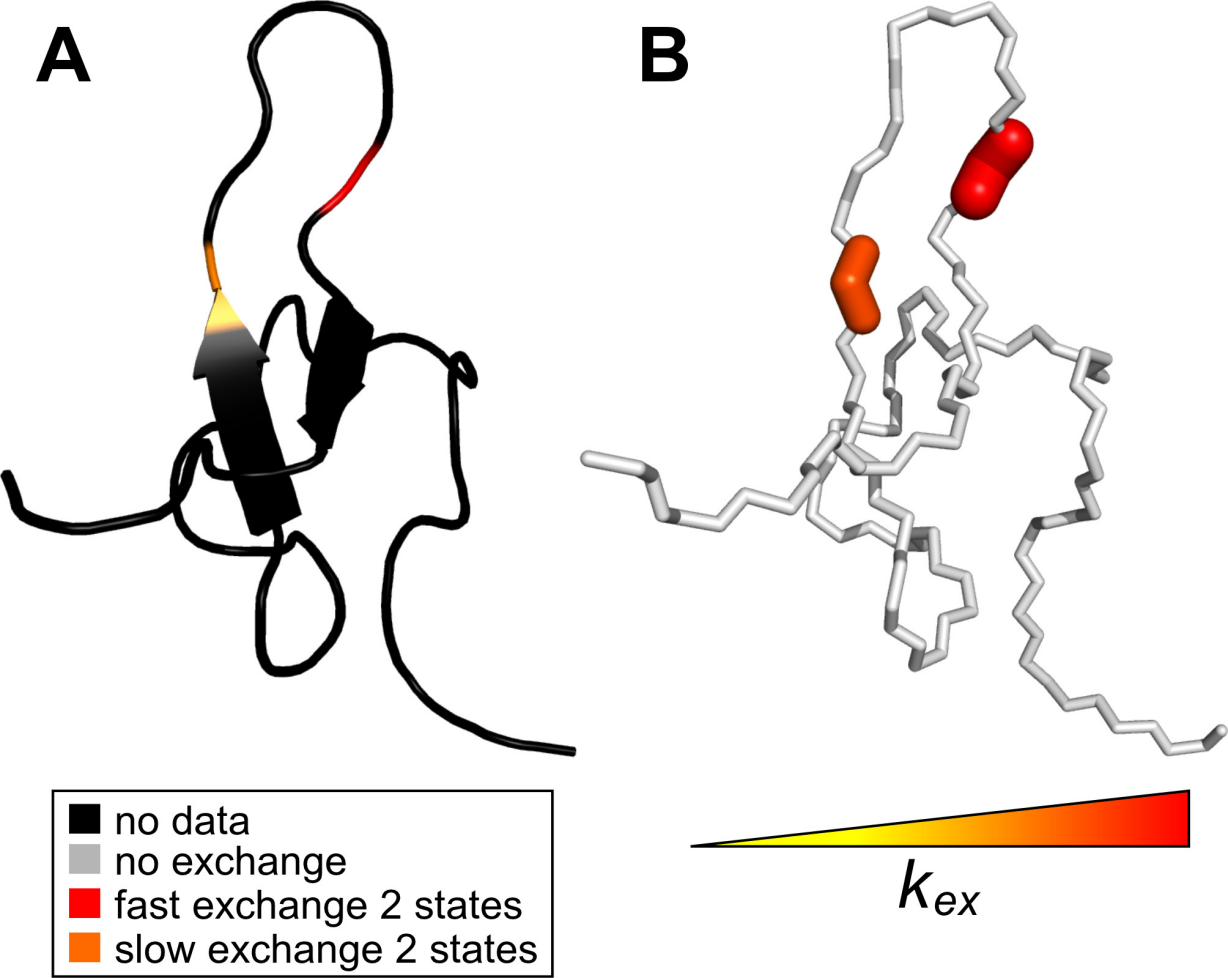
**Color coded structures created by PyMOL macros by NESSY**. PCTX1 structure is color coded according to selected model (A) and *k_ex _*(B). Note only 2 residues were analyzed and therefore, the majority of the structure is colored in black (no data). Different models are colored differently; increasing values are colored from yellow to red and line width is increased for increasing values of k_ex_.

To best illustrate calculated motion, NESSY creates PyMol macros for color and width-coded structures. Such structures are created for model selection, *k_ex _*and *R_ex_*. Figure 4 shows color coding for both model selection and *k_ex _*of PCTX1. Models are visualized in different colors per residue, while extracted dynamics parameters (*k_ex _*and *R_ex_*) are colored from yellow to red and also represented by thicker lines for increasing values. Note that in Figure 4 only two residues were analyzed and therefore, only two residues are colored.

### Global fit to synthetic data

The dynamics parameters, *k_ex_*, *p_b _*and *δω*, are highly interdependent and data from a single static field is typically insufficient for their accurate determination. Consequently acquisition of relaxation dispersion data at two or more fields is necessary for the robust and accurate estimation of these parameters [20]. NESSY is able to perform a global fit by sharing parameters *k_ex_*, *p_b _*and *δω*, but taking the field dependency of *δω *into account. To demonstrate this function, synthetic data was created for data virtually recorded at 800 and 600 MHz. For 800 MHz data, synthetic data (5% error) previously described was used. Additionally, synthetic data was generated at 600 MHz (*δω_600 _*= 600/800 *δω_800_*). Both data sets were simultaneously fitted to models 1 to 3 (Table 3). Model selection was performed using AICc and errors of fit for the parameters were estimated by 500 Monte Carlo simulations per residue and individual experiments.

**Table 3:** Model selection of global fit of synthetic relaxation dispersion data.

Synthetic	Model 1		Model 2		Model 3		Selected
	χ^2^	AICc	χ^2^	AICc	χ^2^	AICc	
		
Model 2	545.3	549.3	9.8	21.8	43.4	57.4	2
Model 3	3434.6	3438.6	126.7	138.6	10.1	24.1	3

**Figure 5:**
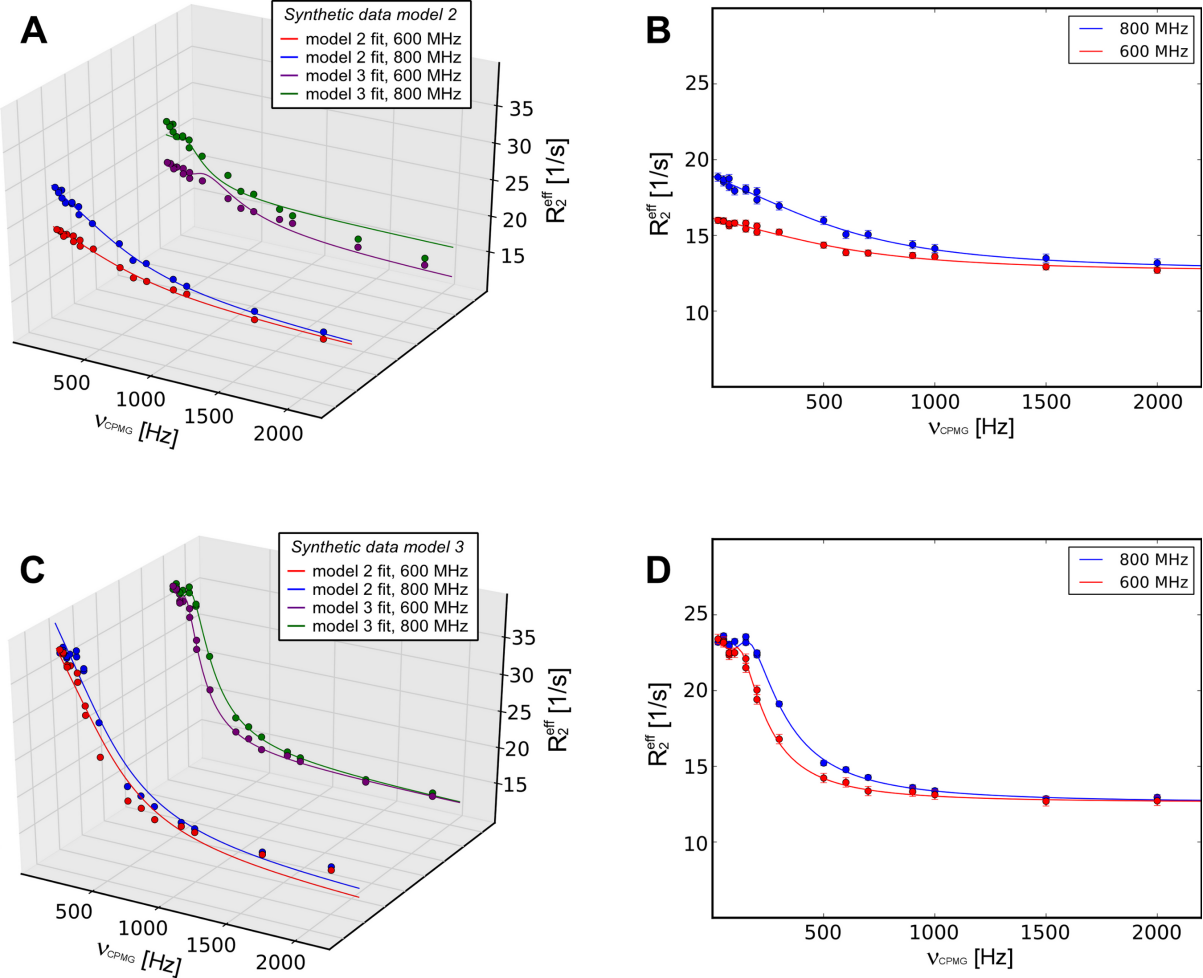
**Global fit for synthetic data at 2 field strengths**. Synthetic data for model 2 (A and B) and model 3 (C and D) were generated for two field strengths: 600 MHz (red and purple) and 800 MHz (blue and green). (A) Model 2 data was simultaneously fitted to models 1 to 3; only fits to models 2 and 3 are displayed. (B) Best selected model (model 2) is separately plotted. (C) Synthetic data of model 3 was fitted to models 1 to 3; only fits to models 2 and 3 are shown. (D) Best selected model (model 3) is separately plotted.

For the synthetic data for model 2 at two different spectrometer frequencies, fits (χ^2^) generally improved with increasing number of parameters. Using AICc model selection NESSY clearly chooses the correct model (model 2). For the synthetic data for model 3, the correctly chosen model according to AICc was model 3 (Table 3). A summary of extracted parameters is given in Table 4 and fits to models 2 and 3 for both synthetic data sets (each at two spectrometer frequencies) are shown in Figure 5.

**Table 4:** Extracted dynamic parameters of global fit of relaxation dispersion data.

Synthetic	Model	R_2 _[1/s]	err	k_ex _[1/s]	err	p_b_	err	δω [rad/s]	err
*Model 2*		*15.23*		*3750.25*					
Fit to Model 2	2	15.24	0.29	3876.90	194.54				
*Model 3*		*15.23*		*306.15*		*0.07*		*1875.51*	
Fit to Model 3	3	15.18	0.09	334.61	60.77	0.07	0.02	1918.98	20.50

### Cluster Analysis

**Figure 6:**
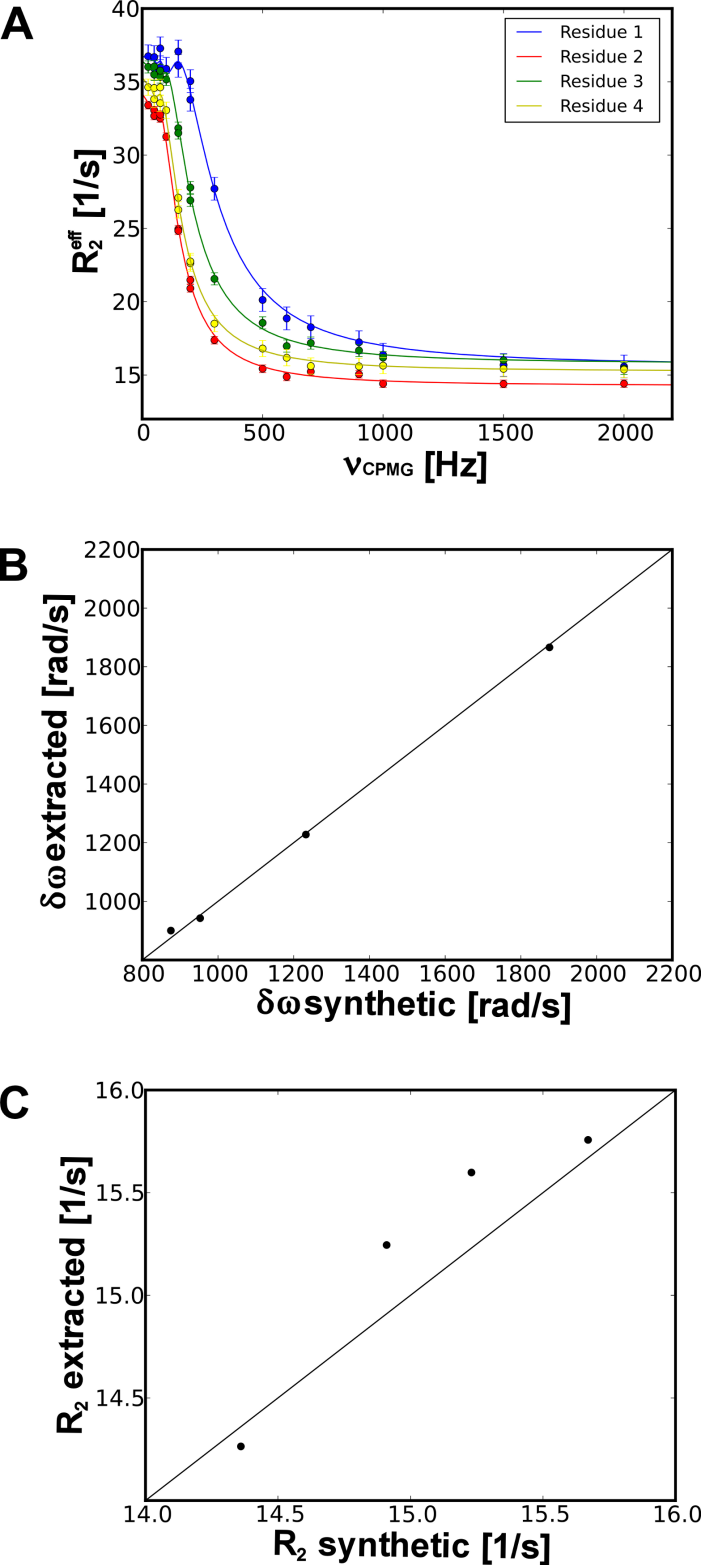
**Cluster analysis**. (A) Synthetic data for 4 residues in 2 state slow exchange (model 3) were created and simultaneously analyzed. (B and C) Correlation plots of extracted versus synthetic δω and R_2_, respectively.

Under certain circumstances, motion in different parts of a macromolecule may be concerted. Therefore, multiple residues/nuclei may experience the same exchange. In such cases, it is useful to cluster these residues and fit them simultaneously. Cluster analysis of data recorded at one or more static fields is included in NESSY, where single values of k_ex _and populations are determined for all residues. Individual parameters, δω and ϕ (eq. 4) are obtained for each residue. To demonstrate this function, synthetic data for 4 residues experiencing k_ex _of 306.15 1/s and a minor population (p_b_) of 0.072 were created using model 3 (2 state slow-limit exchange) consisting of 5% noise. The transverse relaxation rate, $R_{2}^{0}$, as well as δω were different for each residue. The data was clustered and simultaneously fitted to models 2 or 3 (Figure 6A). The selected model by AICc was model 3 and the extracted exchange constant k_ex _was 286.69 ± 66.5 1/s and population p_b _was 0.075 ± 0.013. Furthermore, the extracted values for δω and $R_{2}^{0}$ correlated to their initial values (Figure 6B, C).

## Discussion

The software presented in this article permits the automated data analysis of relaxation dispersion experiments. The user has the opportunity to choose to fit to the entire data set or individually selected residues. In addition, data from two or more magnetic fields can be globally fitted. During global fits, field dependency of *δω *is taken into account. Multiple residues can be grouped and analyzed simultaneously using built-in cluster analysis (individual or global fit).

Maximum flexibility of data entry has been included. For example, peak lists containing peak intensities that are created by any other software can be imported; protein sequences are read either from PDB files, retrieved from the internet using UniProt identifier http://www.expasy.ch or can be added manually; CPMG frequencies are read directly from Bruker VD (variable delay) lists. Furthermore, NESSY is linked to the Bruker Protein Dynamic Center (PDC, starting with version 1.1, http://www.bruker-biospin.com, in collaboration with Dr. Klaus-Peter Neidig) so that projects can be exported in PDC and directly read into NESSY for extended data analysis. In addition, NMRView [21] tables can be imported directly.

During the calculation, NESSY produces plots and CSV files (text files compatible with Excel and OpenOffice) of the $R_{2}^{eff}$ profiles and the individual fits for each calculated residue as well as the extracted values (such as *R_2_*, *k_ex _*and *p_b_*) and statistics (*χ^2 ^*and AICc) for each model. In addition, color coded structures for selected models, *k_ex _*and *R_ex _*are created (structures are drawn using Pymol). Furthermore, NESSY offers tools to create custom made 2D and 3D plots in different formats and styles suitable for publication (see Figures 2 to 6).

Synthetic data as well as experimental NMR CPMG relaxation dispersion data were analyzed using NESSY. The quality of fits of the synthetic data was assessed by comparing original values to those extracted from data analysis (Table 1 and 2, Figure 2). For choosing the best fitting model NESSY evaluates the need and benefit for describing the experimental data with more parameters by using AICc, AIC or F-test. By default, model selection is performed using AICc to avoid over fitting, as more parameters (more complex models) may give better fits. The advantage of using AICc (and AIC) compared to F-test is that data do not have to be normally distributed. As relaxation dispersion experiments usually consist of 15 to 20 data sets, normal distribution of data cannot be assumed. For the synthetic data the correct model was consistently chosen and the extracted values were statistically similar to the initial values. To demonstrate usability of NESSY for NMR data, two signals of PCTX1 were analyzed (Figures 3 and 4). One signal experienced slow-limit and the other fast-limit chemical exchange. In the case of slow exchange, the minor populated conformation was present at 0.8%, which is in accordance with the NMR spectra, where only one peak is visible.

To be able to unambiguously distinguish between fast and slow exchange and to extract populations and shift differences, experiments should be collected at two or more different magnetic fields [20]. NESSY supports global fitting at multiple field strengths, while taking the field dependency of *δω *into account. Synthetic data for models 2 and 3 at two different static frequencies (600 and 800 MHz) were created and analyzed. Data were fitted to models 1 to 3 and in each case the correct model was chosen for both global fits (Tables 3 and 4, Figure 5).

## Conclusions

In this article, the main features of NESSY have been presented, such as curve fitting, model selection and data presentation of relaxation dispersion experiments. Nevertheless, NESSY is not limited to these functions. As relaxation dispersion experiments enable the extraction of populations of individual states, NESSY offers tools to calculate the free energy (ΔG) between states. For data that fit best to fast exchange models, the populations of each state cannot be extracted, as *k_ex _*and *δω *cannot be uniquely determined. An integrated function in NESSY is to calculate the populations for residues with known chemical shift differences, such as observed in ligand binding experiments. If a series of experiments are recorded over a temperature range, the temperature dependence of ΔG can be used to extract entropy (ΔS) and enthalpy (ΔH) changes using van't Hoff analysis. NESSY supports automatic van't Hoff analysis of both linear and non-linear models [22]. Furthermore, NESSY can calculate activation energy barriers using transition state theory and the Eyring equation and generate energy landscape plots.

Taken together, we present user-friendly software for NMR relaxation dispersion (^15^N/^13^C) data analysis that requires minimal user intervention. In addition, NESSY can be used to analyze biophysical experiments, such as van't Hoff and transition state theory analysis and to create publication quality 2D and 3D plots. Due to its flexibility, users can choose between different relaxation dispersion models and statistical tests for model selection. Results generated by NESSY are aimed to be usable for publication. Tables can be produced directly using CSV files; 2D and 3D plots are created during or after analysis and color coded structures can be directly used in publications. Note that each figure display in this article was created in NESSY. This software is open source and freely available from http://nessy.biochem.unimelb.edu.au. NESSY comes with a detailed manual and tutorial. For additional help, questions can be addressed at the NESSY mailing list (nessy-users@gna.org).

## Availability and requirements

• **Project name: **NESSY

• **Project home page: **http://nessy.biochem.unimelb.edu.au

• **Operating system(s): **Platform independent

• **Programming language: **Python

• **Other requirements: **Scipy, Numpy, wxPython, Matplotlib (None for compiled binaries)

• **License: **GNU GPL v3

• **Any restrictions to use by non-academics: **None

## Abbreviations

AIC: Akaike's Information Criteria; AICc: Akaike's Information Criteria with second order correction for small sample size; CPMG: Carr-Purcell-Meiboom-Gill; NESSY: NMR Relaxation Dispersion Spectroscopy Analysis Software; NMR: Nuclear Magnetic Resonance; NOE: Nuclear Overhauser Effect.

## Competing interests

The authors declare that they have no competing interests.

## Authors' contributions

MB wrote the code, conducted the NMR experiments and data analysis and wrote the manuscript. PRG supervised the project, assisted in acquisition and processing of NMR experiments and wrote the manuscript. All authors read and approved the final manuscript.
